# Extra-striatal Uptake of ^99m^Tc-TRODAT SPECT in a Cerebral Meningioma: A Case Report

**DOI:** 10.22038/AOJNMB.2018.11933

**Published:** 2019

**Authors:** Mahsa Sabour, Ali Shoeibi, Somayeh Ghahremani, Ramin Sadeghi

**Affiliations:** 1 Nuclear Medicine Research Center, Mashhad University of Medical Sciences, Mashhad, Iran; 2 Department of Neurology, Faculty of Medicine, Mashhad University of Medical Sciences, Mashhad, Iran

**Keywords:** Meningioma, Nuclear medicine, Parkinsonism, TRODAT

## Abstract

We reported a 71 years old woman, with history of rest and postural tremor, bradykinesia and memory problems. In her dynamic MRI, a contrast-enhanced tumor in the cerebellopontine (CP) angle was found which was compatible with a meningioma. ^99m^Tc-TRODAT SPECT showed decreased activity in the left putamen, indicating idiopathic Parkinson disease. There was also a focus of increased activity on the right side of the skull base, which was compatible with meningioma in MRI.

## Introduction

^99m^Tc-TRODAT scintigraphy is a useful method for diagnostic approach in parkinsonism. This tracer is a ligand which specifically binds to Dopamine transporters (1). In addition to basal ganglia, uptake of the DAT derivatives in other brain pathologies can be seen which should not be mistaken with the normal basal ganglia uptake. In the current case report, ^99m^Tc-TRODAT uptake in a CP angle meningioma is reported.

## Case report

**Figure 1:**
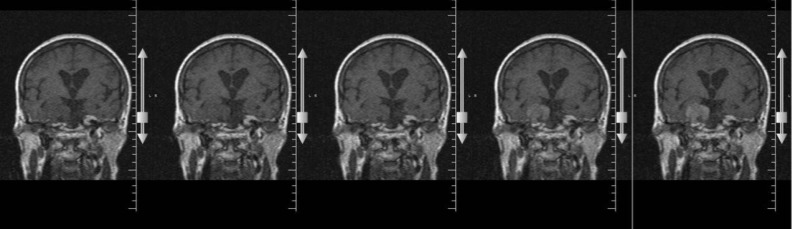
Contrast (Gadolinium) enhanced MRI (T2-weighted) of the patient which shows a CP angle tumor with enhancement compatible with meningioma

Our case was a 71 years old woman suffering from rest and postural tremor in the upper limbs since 8 years ago. She also complained of bradykinesia and memory problems. In her dynamic MRI (Siemens, Germany, T2-weighted with Gadolinium enhancement), a contrast-enhanced tumor in the cerebello-pontine (CP) angle was found which was compatible with a meningioma (Figure 1). 

**Figure 2:**
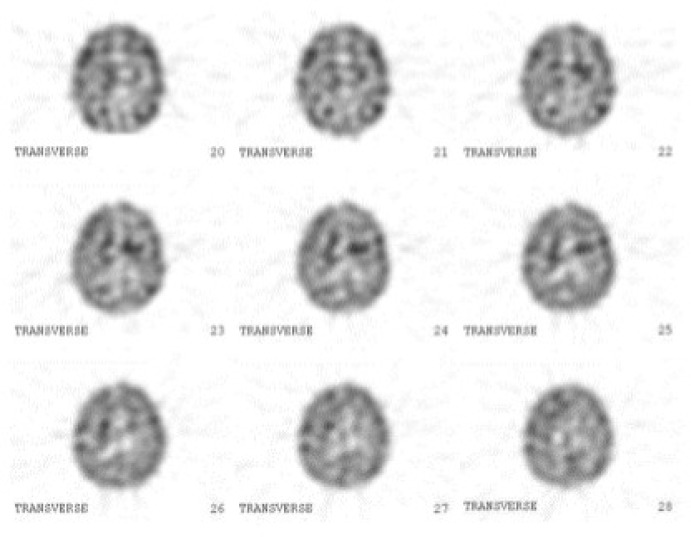
**. **Reconstructed ^99m^Tc-TRODAT images which showed decreased uptake in the basal ganglia

For differentiation of idiopathic Parkinson disease from essential tremor, dopamine transporter study with ^99m^Tc-TRODAT-1 was requested. 4 hours after intravenous administration of 20 mCi (740 MBq) of ^99m^Tc-TRODAT-1, brain SPECT was obtained using a dual head gamma camera (ADAC, USA) equipped with low energy high resolution collimator. Data acquisition was performed in matrix size of 128×128 and 360^ο^ arc (180^ο^ for each head) with 64 projections and 30 seconds per projection. Reconstruction was done with Butterworth filter with cut off frequency of 0.35 and order of 10. Chang method was used for attenuation correction. Reconstructed SPECT images showed decreased radiotracer uptake in the left putamen compatible with idiopathic Parkinson disease (Figure 2). 

**Figure 3:**
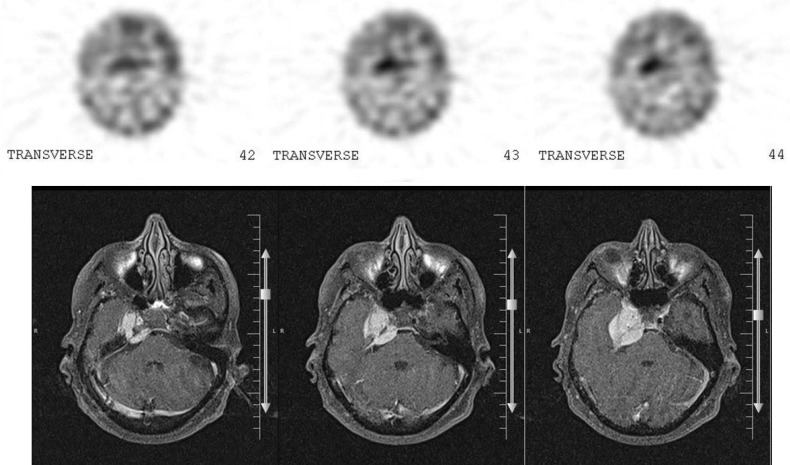
Reconstructed ^99m^Tc-TRODAT images as well as the corresponding MRI (T2-weighted) which show the tracer uptake in the meningioma (MRI and SPECT images both were presented parallel to the orbito-meatal line).

There was also a focus of increased activity on the right side of the skull base (right CP-angle), which was compatible with meningioma on MRI. Tumor to cerebellum count ratio was 7.8 on reconstructed SPECT images (Figure 3).

## Discussion

Parkinson disease (PD) is one of the most common causes of movement disorders, which accounts for 2% of population over 65 years old (2). It usually presents with rest tremor, bradykinesia and rigidity (3). Diagnosis is generally based on clinical signs and symptoms, but overlap of these findings can cause serious misdiagnosis (4). Anatomical imaging modalities such as computed tomography (CT) and (MRI) show abnormality in advanced stages of PD (2). Also, they can be used for excluding probable underlying causes of Parkinsonism. Some other conditions like structural, toxic, metabolic and infectious pathologies can cause secondary Parkinsonism (3). Cerebral tumors are rare but important causes of parkinsonism (5) and meningiomas are the most common (3). Meningiomas are non-glial tumors with annual incidence of 6 per 100,000 population. According to WHO classification, majority of lesions are grade I (benign) and more aggressive subtypes are grade II (atypical) and grade III (anaplastic) (6). Most meningiomas have some characteristic features in MRI including an extraaxial mass which is isointense relative to normal grey matter in T1 and T2 sequences and after gadolinium administration avid homogenous enhancement is seen (7). 

Functional imaging may help to differentiate idiopathic Parkinson disease from other causes of movement disorders. Using specific dopamine radiotracers can assess the nigrostriatal pathway and degeneration of dopaminergic neurons in substantia nigra which is the cardinal pathogenesis in PD (8). In dopamine transporter (DAT) studies, asymmetric reduction of tracer binding in striatum, especially in the putamen contralateral to the parkinsonian symptoms is seen in idiopathic PD, whereas this study is normal in essential tremor. Variable findings can be seen in DAT scan in secondary Parkinsonism (3). According to new movement disorder society (MDS) criteria, normal functional neuroimaging study of dopaminergic system can rule out the Parkinson disease (9). ^99m^Tc-TRODAT-1 scintigraphy is also useful for assessing the disease progression (10). 

Extra-striatal uptake of ^99m^Tc-TRODAT was reported in some cerebral tumors like meningioma, oligodendroglioma, clival tumors and metastasis as well as subdural hematoma (9). 

Our case is one of a few cases of meningioma uptake in ^99m^Tc-TRODAT-1 SPECT. Yu-Li Chiu et al and Taise Vitor et al reported incidental finding of brain meningiomas in ^99m^Tc-TRODAT-1 scintigraphy (11, 12). Also, Piush Chandra reported extrastriatal uptake of meningioma in ^99m^Tc-TRODAT-1 SPECT/CT (9). Our case shows the importance of association of anatomical and functional imaging in assessing Parkinsonism.
